# Effects of Grape Seed Proanthocyanidins and Malic Acid on Digestive Characteristics of Starch in Bread

**DOI:** 10.3390/foods15010149

**Published:** 2026-01-02

**Authors:** Xinguang Qin, Qinyue Zhu, Guanxi Li, Haizhi Zhang, Xiaohui Di, Liang Liu, Gang Liu, Andreas Blennow

**Affiliations:** 1 College of Food Science and Engineering, Wuhan Polytechnic University, Wuhan 430023, Chinahzzhang@iccas.ac.cn (H.Z.); liugang1982@whpu.edu.cn (G.L.); 2 School of Life Science and Technology, Wuhan Polytechnic University, Wuhan 430023, China; hustll@126.com; 3 Department of Biotechnology and Food Engineering, Guangdong Technion—Israel Institute of Technology, Shantou 515063, China

**Keywords:** grape seed proanthocyanidins, malic acid, bread, starch digestibility, α-amylase inhibition

## Abstract

The effects of grape-seed proanthocyanidins (GSP) and malic acid (MA) on the multiscale structure and digestibility of starch in a bread model were investigated. Fourier transform infrared (FTIR), Raman spectroscopy analyses, long-range order (crystallinity), amylolytic release of glucose, as well as the effect on α-amylase activity of starch in the bread, were determined. The combination of GSP and MA increased the molecular order but decreased the crystallinity of the starch. Amylase fluorescence spectra showed that the α-amylase was notably quenchable by adding GSP and MA, and the inhibition rate of α-amylase reached 10.3%. Confocal Laser Scanning Microscopy (CLSM) imaging confirmed the digestion data in vitro showing that in the presence of 0.3% GSP and 0.5% MA in the bread, the glucose release of the bread was reduced to 5.43%. These findings demonstrate that GSP and MA can effectively modulate starch structure and digestibility in bread, offering a strategy to control glucose release in baked foods.

## 1. Introduction

The relentless pace of daily human life and the resulting stress have significantly increased the incidence of diabetes and obesity, which are chronic metabolic diseases [1]. High carbohydrate uptake may cause the postprandial blood sugar levels to fluctuate sharply, posing a potential health threat. Bread is one of the most widely consumed grain products in global staple diets, particularly occupying a prominent position in the daily carbohydrate intake of consumers in Western countries. As a high-carbohydrate food, bread is rich in starch, the digestion rate and extent of which affect human health [2]. The starch gelatinization process during baking forms readily absorbable structures, making bread an ideal model for manipulating starch digestion rates and mitigating postprandial blood glucose fluctuations [3].

The content of grape-seed proanthocyanidins (GSP) is very rich. Proanthocyanidins can be non-covalently bound to starch through hydrogen bonding. Accordingly, phenolic substances enter the inner cavity of starch helical structures to form a starch–polyphenol complex, thereby modulating starch digestion and improving the physicochemical properties of starch [4]. Anthocyanins are an important component associated with starch digestion through the ordered aggregation structure of the starch. Various dietary sources of anthocyanins efficiently inhibit α-glucosidase and α-amylase activities, which delay starch digestion in the human body [5,6,7,8,9]. This delay effectively delays the time for glucose to enter the bloodstream, substantially reducing postprandial blood glucose levels [10]. Previous data documented the inhibitory mechanisms of anthocyanins of α-amylase and α-glucosidase and details some of the anthocyanin–dough interactions [11]. However, procyanidins are readily destroyed during food processing and storage by various factors, including pH, light, and temperature [12]. High temperature and extended baking times typically lead to notable loss of proanthocyanidin content [13]. Proanthocyanidin stability in fruit juices, beverages, and biscuits, etc., has been explored [14], but research on enhancing proanthocyanidin stability in bread systems, including interaction with starch and high-temperature baking, is limited.

Malic acid (MA), as an intermediate product of the tricarboxylic acid cycle, is an important component in human metabolism [15]. MA and starch can be esterified to produce MA-esterified starch at high temperature. This esterified starch has high resistance to enzymatic hydrolysis [16] and can increase the amount of health-promoting resistant starch in the system, thereby delaying the rise in postprandial blood sugar.

The hydroxyl group on the anthocyanin structure can combine with the ester bonds of one or several molecules of organic acids or flavonoids to form acylated anthocyanins, thereby improving anthocyanin stability. The enhanced retention of anthocyanins in blue corn cookies has been investigated using different acidifiers and acidic substances [17], and that in brews made from freshly picked Kenyan TRFK 306 tea leaves has also been studied through a combination with citric acid [18]. The color intensity and thermal stability of tart cherry anthocyanins can reportedly be improved by tannic acid, caffeic acid, gallic acid, and MA [19]. However, no study has focused on the effects of combining MA and GSP on the digestive characteristics of bread.

In the current study, the combined influence of GSP and MA on the structure of flour starch and the digestive characteristics of bread was investigated. The fluorescence-quenching effects of GSP and MA on digestive enzymes were studied. Changes in the microstructure and glucose release of bread at different digestion stages were then investigated by simulated in vitro digestion.

## 2. Materials and Methods

### 2.1. Experimental Materials

GSP (≥95% purity) was obtained from Shaanxi Jinrun Biotechnology Co., Ltd (Xi’an, China). MA (analytical grade; ≥99.5% purity) was purchased from Shanghai Yuanye Biotechnology Co., Ltd (Shanghai, China). Wheat flour (Golden Image brand) was purchased from Jiangsu Nanshun Food Co., Ltd (Changzhou, China). All other chemicals were of analytical-reagent grade, and ultrapure water was used.

### 2.2. Separation of Wheat Starch

Ten grams of wheat flour was mixed with 0.1%, 0.3%, and 0.5% GSP (*w*/*w*). To these mixtures, 0.1%, 0.3%, and 0.5% MA were added to the 0.3% GSP sample at three different concentrations. The samples were denoted as 0.3% GSP-0.1% MA, 0.3% GSP-0.3% MA, and 0.3% GSP-0.5% MA, respectively. Gluten and starch were separated and prepared with a gluten analyzer (2200, Perten Instruments AB, Stockholm, Sweden). 4.8 ± 0.4 mL of sodium chloride solution was used to evenly distribute the solution on the surface of the flour before washing. Washing was performed until the effluent from the washing chamber was clear. This washing liquid was starch paste, which was centrifuged at 4000× *g* rpm for 15 min (T-18, IKA Works GmbH & Co., Staufen, Germany). The precipitate was collected for freeze-drying. After drying, the material was passed through a 200-mesh sieve for use.

### 2.3. Fourier Transform Infrared (FTIR) Spectroscopy Analysis

FTIR spectroscopy analysis (Frontier, PerkinElmer, Inc., Waltham, MA, USA) was performed according to a previous method [20]. One mg of starch sample was accurately weighed and uniformly mixed with 100 mg of KBr powder, pressed into a tablet, and incubated at room temperature for 5 min. Detection was performed with a DLATGS detector at 4 cm^−1^ resolution over a 400–4000 cm^−1^ wavelength range and with 15 scans.

### 2.4. Raman Spectral Analysis

The previously described method was adopted with minor modifications [21]. The instrument used was a Raman spectrometer (inVia, Renishaw, UK). A small amount of starch was laid flat on a slide, and samples were collected within the range of 500–4000 cm^−1^ using a 633 nm excitation wavelength, 10 mW laser power, and 10 scans. Full-width at half-maximum (FWHM) values were calculated.

### 2.5. Wide Angle X-Ray Scattering (WAXS) Analysis

Based on a previous method [22], the crystal structure of starch with different GSP and MA contents was characterized by WAXS (SmartLab, Rigaku, Japan). A starch powder sample (20 mg) was equilibrated to 50% moisture and subjected to X-ray irradiation using a Cu–Kα radiation source. The sample was scanned at 4°/min, 40 kV, and 40 mA. Jade 6.0 was used for data processing.

### 2.6. Bread Preparation

Bread was prepared according to Begum et al. [23] with minor modifications. The bread recipe comprised 200 g flour, 2.8 g yeast, 20 g sugar, 120 g water, 10 g butter, and 1.2 g salt. Proanthocyanidins and malic acid were added at ratios of 0.1%, 0.3%, and 0.5% (by flour weight). Flour, sugar, and yeast were mixed in a mixer (SP-12, Jiasheng Technology Co., Ltd., Hong Kong, China) at low speed for 2 min. Water was added, and mixing continued for 3 min. Butter was incorporated, and the mixture was beaten at high speed for 2 min until satisfactory gluten development. Salt was added, and mixing continued for 3 min until the dough surface became smooth, concluding the kneading process. The dough was covered with plastic wrap, and fermented for 30 min (85% humidity, 30 °C), thereafter divided into 40 g portions, shaped and degassed with a rolling pin, covered with plastic wrap, and placed in a constant temperature and humidity chamber (85% humidity, 30 °C) for a 60 min fermentation. The fermented dough was baked at 190 °C on the upper rack and 200 °C on the lower rack for 12 min. After cooling for 2 h, the bread was stored in resealable bags until analysis. The control group is bread without GSP and MA added.

### 2.7. Bread Simulates Digestion In Vitro

Bread samples were digested using an oral and gastro-small intestine three-phase static in vitro digestive system in vitro as described [7]. The three types of fluids used were as follows: simulated salivary fluid (SSF), simulated gastric fluid (SGF), and simulated intestinal fluid (SIF). They comprised the corresponding electrolyte stock solutions and proteins. Bread and water were homogenized for 2 min at a ratio of 1:3 to enable even distribution of bread samples. The specific test procedure was as follows.

In the oral stage, 5 g of bread samples was initially mixed with an equal volume of the digestive solution, including SSF and 25 μL of CaCl_2_ (0.3 M). α-amylase (75 U/mL) and water were added to a final volume of 10 mL. The pH was adjusted to 7.0.

In the gastric stage, 10 mL of oral digesta was mixed with SFG at a 1:1 (*v*/*v*) ratio. Pig pepsin was added so that the final enzyme activity in the solution reached 2000 U/mL. Following the addition of 5 µL of CaCl_2_, pH was adjusted to 3.0 with 1 mol/L HCl. The reaction was performed at 37 °C for 2 h. Aliquots (2 mL) of digesta were collected at 0, 1, and 2 h, respectively, rapidly centrifuged at 4 °C for 10 min (5000× *g*), and the supernatant was frozen at −20 °C prior to analysis.

For the intestinal stage, 20 mL of oral digesta was mixed with SIF at a 1:1 (*v*/*v*) ratio. The final enzyme activity of pancreatic enzyme was 100 U/mL, the enzyme activity of saccharifying enzyme was 1.3 U/mL, and 40 μL of (0.3 M) CaCl_2_ was added. The pH was adjusted with 1 mol/L NaOH to 7.0, and the reaction was incubated for 3 h at 37 °C with gentle stirring. 500 μL aliquots were withdrawn at 0, 30, 60, 90, 120, 150, and 180 min and mixed with 4500 μL of ethanol. The samples were centrifuged at 4000× *g*, and the supernatant was collected. The glucose concentration was determined using 3,5-dinitrosalicylic acid colorimetry (DNS).

### 2.8. Confocal Laser Scanning Microscopy

Based on a previous method [24], confocal laser scanning microscopy (CLSM, OLYMPUS FV 1200, Malvern, Japan) was used to observe the changes in starch microstructure during digestion. Equal volumes of mouth, stomach, and intestinal digesta were incubated for 5 min in boiling water and centrifuged at 15,000× *g* for 10 min. The precipitate was collected and analyzed. Fluorescein isothiocyanate FITC (0.02%) and Rhodamine B (0.02%) were mixed in a ratio of 1:1 and used for the detection of starch and gluten protein, respectively. Dyeing was performed for 2 min, and excess dye was washed off. The digestive fluid was observed using a 40× magnification objective lens. FITC and Rhodamine B were excited at 488 and 533 nm, respectively.

### 2.9. Inhibitory Activity of Bread Extracts on Digestive Enzymes

(1) α-Amylase

The inhibitory activity of digestive enzymes was monitored as described [25]. Typically, 25 μL of extract was mixed with 25 μL of α-amylase solution (0.5 mg/mL, 20 mM sodium dihydrogen phosphate solution, pH 6.9). Following incubation at 37 °C for 10 min, 25 μL of starch solution (0.5%, gelatinized at 80 °C for 30 min) was added, and DNS (50 μL) was added. The mixture was incubated at 100 °C for 5 min, terminated by cooling, and directly measured at 540 nm.

(1) $$\alpha -amylase\;lnhibition\;ratio=\left(1-\frac{A_{3}-A_{2}}{A_{1}-Ao}\right)\times 100$$

where A_3_: α-amylase solution and sample; A_2_: without α-amylase solution; A_1_: without no samples; and A_0_: neither α-amylase nor sample.

(2) α-Glucosidase

Extract (25 μL) was mixed with 25 μL of α-glucosidase solution (1 U/mL in 0.1 M phosphate buffer, pH 6.9), incubated for 10 min at 37 °C, and 25 μL of 5 mM PNPG solution was added. The mixture was incubated for 10 min at 37 °C and finally, 50 μL of 100 mM sodium carbonate solution was added to terminate the reaction. Absorbance was measured at 450 nm against water.

(2) $$\alpha -\mathrm{glucosidase}lnhibition\;ratio=\left(1-\frac{A_{3}-A_{2}}{A_{1}-Ao}\right)\times 100$$

where A_3_: α-glucosidase and sample added; A_2_: no α-glucosidase; A_1_: no sample; and A_0_: neither α-glucosidase nor sample.

### 2.10. Effect of Bread Extract on the Fluorescence Intensity of Digestive Enzymes

Enzyme fluorescence of α-glucosidase and α-amylase was analyzed with a fluorescence spectrophotometer (F-4600, Hitachi, Tokyo, Japan) [26]. Sample extract (0.2 mL) was mixed with 3 mL of α-amylase and α-glucosidase solutions, the volume fixed to 10 mL with water, and the mixture incubated for 30 min at 37 °C. The samples were excited at 278 nm in triplicate, the emission was measured at 290 nm, and the scanning range was 290–500 nm.

### 2.11. Statistical Analysis

All experiments were repeated at least in triplicate, and data are expressed as the mean ± standard deviation. SPASS 17.0 single-factor ANOVA was used to evaluate the difference between the mean values. *p* < 0.05 indicates a significant difference.

## 3. Results and Discussion

### 3.1. Fourier Transform Infrared Spectroscopy Analysis of Wheat Starch by Proanthocyanidins Combined with MA

**Figure 1:**
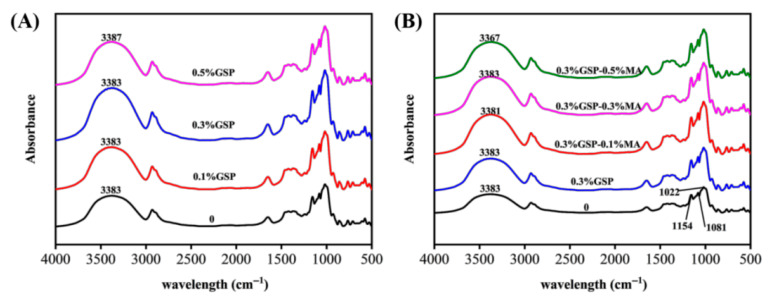
FTIR spectra of starch. (**A**) Exclusion of GSP from starch and addition of 0.1%, 0.3%, and 0.5%GSP. (**B**) Addition of 0.3% GSP with 0.1%, 0.3%, and 0.5% MA to the starch.

FTIR is a suitable method for analyzing molecular surface structure, including hydrogen bonding [27]. The presence of GSP and MA did not produce any new distinct peak (Figure 1). However, a strong broad absorption peak at 3400 cm^−1^, associated with O–H stretching vibration, was observed. The O–H peak did not change notably following the addition of GSP, whereas MA caused the O–H peak to shift from 3383 cm^−1^ to 3381 and 3367 cm^−1^. This progressive shift suggests a strengthening of intermolecular hydrogen bonding, likely due to the introduction of additional carboxyl and hydroxyl groups from MA that participate in H-bonded networks with starch hydroxyl groups.

A high ratio of absorption–peak intensities at (1047/1022) cm^−1^ and (1022/995) cm^−1^ (Table 1) indicates a higher degree of molecular order. The increase from 0.877 to 0.886 in the 1047/1022 cm^−1^ ratio in the presence of 0.3% GSP and MA indicated strengthened short-range molecular order. This may stem from hydrogen bonding between GSP hydroxyl groups and starch hydroxyl groups, as well as esterification reactions induced by MA at baking temperatures. However, at the higher 0.5% GSP concentration, this ratio returned to control levels. This reversal likely resulted from excessive proanthocyanidins aggregating or precipitating during mixing or baking, thereby diminishing their effective interaction with starch chains [28]. The nonlinear response emphasizes the importance of optimal bioactive dosage: insufficient amounts yield negligible effects, while excess may cause phase-separated self-aggregation rather than starch binding. Although short-range ordered structures typically obstruct amylolytic catalysis by steric hindrance [29], the limited correlation with digestibility (as shown in Section 3.4) indicated that other structural factors dominate glucose release kinetics [30].

### 3.2. Raman Spectral Analysis of Wheat Starch by Proanthocyanidins Combined with MA

**Figure 2:**
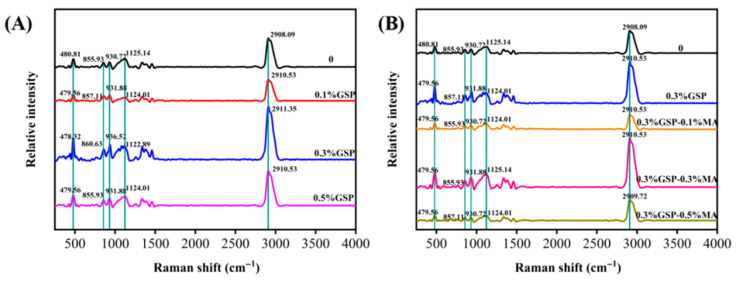
Raman spectra of the starch with different contents of GSP and MA. (**A**) Effects of the addition of 0.1%, 0.3%, and 0.5% GSP in the absence of GSP. (**B**) The presence of 0.1%, 0.3%, and 0.5%MA including 0.3% GSP. Abbreviations as in Figure 1.

The Raman spectrogram of starch (Figure 2) was divided into four regions: <800, 800–1500, 2800–3000 cm^−1^ (C-H stretching), and 3000–3600 cm^−1^ (O-H, very weak). A strong peak value was observed near 2908 cm^−1^, caused by the C-H stretching vibration of starch molecules. The peak at 1125 cm^−1^ is associated with C-O bending vibration. An obvious symmetric stretching vibration peak appeared at 930 cm^−1^, which is caused by the C-H stretching vibration. The peak at 1125 cm^−1^ is associated with C-O bending vibration. An obvious symmetric stretching vibration peak appeared at 930 cm^−1^, caused by an α-1,4-glucoside bond (C-O-C) skeleton vibration. The peak at 856 cm^−1^ is linked to the deformation vibration of an α-1, 4-glucoside bond and the C-C skeleton bonds. The peak at 480 cm^−1^ is associated with the skeleton vibration peak of the C-C-C pyran ring, characteristic of starch [31].

The full width at half-maximum (FWHM) of the Raman spectrum at 480 cm^−1^ can represent the structural alternations of the starch matrix. FWHM decreased from 29.09 for the control group to 27.15 in the presence of 0.3% GSP (Table 2). Such an effect is possibly due to GSP forming hydrogen bonds or hydrophobic stacking interactions with helical segments of glucan chains. However, when the GSP concentration reached 0.5%, FWHM increased to 29.63, indicating weakened interactions at high concentrations. This may result from GSP precipitation or steric hindrance effects during dough mixing and baking. In contrast, 0.5% MA combined with 0.3% GSP similarly increased FWHM to 29.52, likely due to partial starch hydrolysis induced by MA during thermal processing [32]. Organic acids can catalyze glycosidic bond cleavage at high temperatures, leading to segment separation and disruption of helical structural continuity. Thus, it can be concluded that a low concentration of MA enhances order through esterification or hydrogen bonding, while excessive MA may produce an opposite depolymerization effect.

### 3.3. Crystal Structures of Wheat Starch with Proanthocyanidins and MA

**Figure 3:**
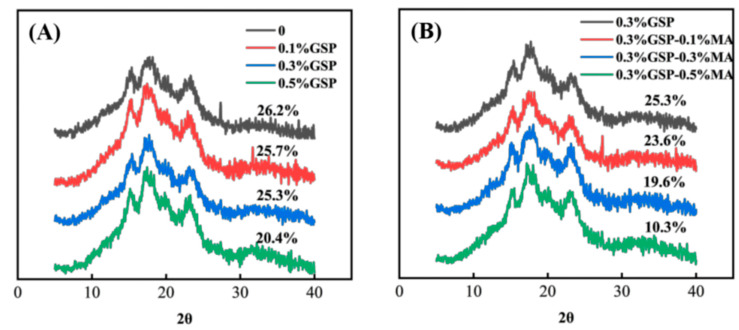
WAXS X-ray diffraction pattern of starch. (**A**) Absence of GSP and presence of 0.1%, 0.3%, and 0.5% GSP. (**B**) Presence of 0.3% GSP with 0.1%, 0.3%, and 0.5% MA. Abbreviations as in Figure 1.

WAXS patterns exhibited characteristic peaks at 2θ = 15°, 17°, 18°, and 23°, with a faint V-shaped peak near 2θ = 20° (Figure 3), confirming the A-type crystalline nature of wheat starch. With the addition of GSP and MA, the relative crystallinity decreased progressively from 26.2% in the control group to 20.7% in the 0.5% GSP group, which may be caused by the disordered transformation of proanthocyanidins and starch molecular chains [33]. The combination of GSP and MA further reduced the crystallinity of starch, primarily because GSP and MA synergistically reduce starch crystallinity. This effect stems from MA-mediated acid-catalyzed hydrolysis, disrupting the double-helix stacking of amylopectin, while the abundant hydroxyl groups in GSP interact with wheat starch to inhibit starch chain rearrangement, jointly preventing the formation of an ordered starch structure [34]. Their synergistic action degrades starch molecular chains and changes the orientation, ordering, and tightness of the starch double helical segments. Ultimately, the double-helix structure became irregularly arranged or even unwound. Consequently, the relative crystallinity decreased [35]. This finding agreed with that of Tian et al. [35], who found that different MA substitution degrees greatly affect the crystallinity of cornstarch.

Figure 3 shows that the characteristic diffraction peak intensity of starch was lower in the presence of GSP and MA, and the A-type crystalline polymorph decreased, which may be due to the interaction between proanthocyanidins and starch, inducing disorder of starch molecular chains in the crystalline region of the starch semi-crystalline layer structure. As a result, the original A-type crystalline polymorph of starch was disrupted. Moreover, the degradation of starch molecular chains by MA disrupted the original hydrogen bonds between molecular chains in starch particles, changed the double-helix orientation of starch chains, the ordered arrangement, and the compactness degree of the double-helix structure, and even the double-helical motifs in the crystalline zones. As a consequence, the A-type crystalline polymorph of starch was reduced [36].

### 3.4. Digestive Characteristics of Bread by Proanthocyanidins Combined with MA

**Figure 4:**
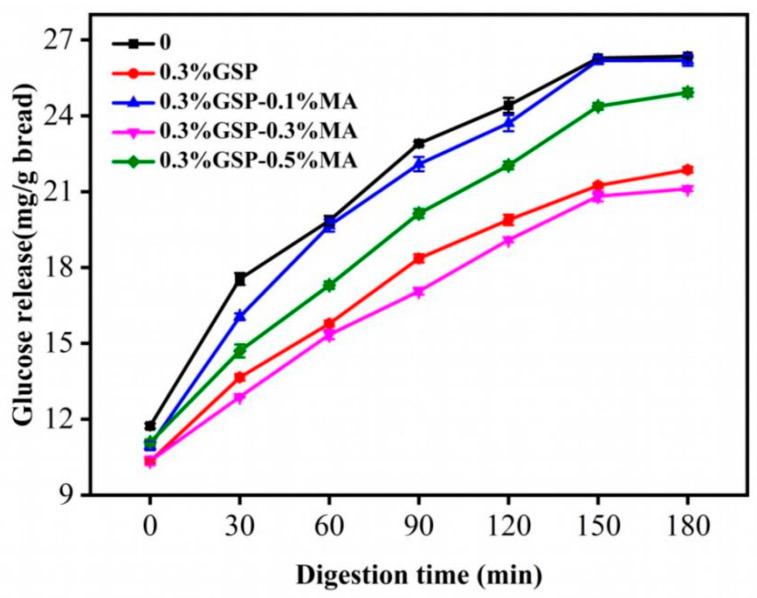
Glucose release rates of raw wheat starch, 0.3%GSP, 0.3%GSP–0.1% MA, 0.3%GSP–0.3% MA, and 0.3%GSP–0.5% MA bread during digestion. Abbreviations as in Figure 1.

The amylolytic glucose release curve of bread during digestion (Figure 4) shows that the control group reached the highest glucose release level, 11.8%, at 0 min. Starch was continuously hydrolyzed, and at the end of digestion, the glucose release of bread supplemented with 0.3%GSP, 0.3%GSP-0.1%MA, 0.3%GSP-0.3%MA, and 0.3%GSP-0.5%MA decreased by 17.0%, 0.6%, 19.8%, and 5.4%, respectively, compared with the control group. In the presence of GSP, the glucose release at the end of the digestion decreased from 26.9% to 22.3% compared with the control group. Hence, GSP significantly inhibited the glucose release during bread digestion. This finding may be due to the GSP-induced enhancement of gluten strength and interference with the interaction between gluten protein and starch. Starch particles are well encapsulated in the gluten structure and are not readily hydrolyzed by amylases [37]. GSP may also delay starch digestion through the direct inhibition of α-amylase activity [38]. Low concentrations of MA reduce digestibility by enhancing the orderliness of starch molecules and synergistically inhibiting α-amylase activity with GSP. Conversely, high concentrations of MA excessively acidify the dough, weakening the dispersion stability of GSP and promoting its localized aggregation and precipitation. This reduces the interaction between effective GSP and starch, partially offsetting its anti-digestive effect.

The glucose release of bread in the presence of GSP and MA changed with different MA concentrations. The 0.3% GSP–0.1% MA group exhibited a release rate of 26.1%, showing no significant difference from the control group. In contrast, the 0.3% GSP–0.3% MA combination demonstrated the highest inhibitory effect on glucose release, reducing the release rate to 20.5%. Organic acids in the gut release H^+^, lowering the pH of the local microenvironment to inhibit amylase activity and delay glucose transmembrane transport. Simultaneously, some organic acid anions (R-COO^−^) can be absorbed into the interstitial fluid via monocarboxylate transporters, participating in the body’s acid-base balance regulation. It was reported that the alkaline microenvironment of the interstitial fluid may facilitate the improvement of glucose response [39], which was further strengthened by the synergistic effect of GSP and MA, jointly leading to a decrease in glucose release rate.

### 3.5. Confocal Laser Analysis of Digestive Fluid

**Figure 5:**
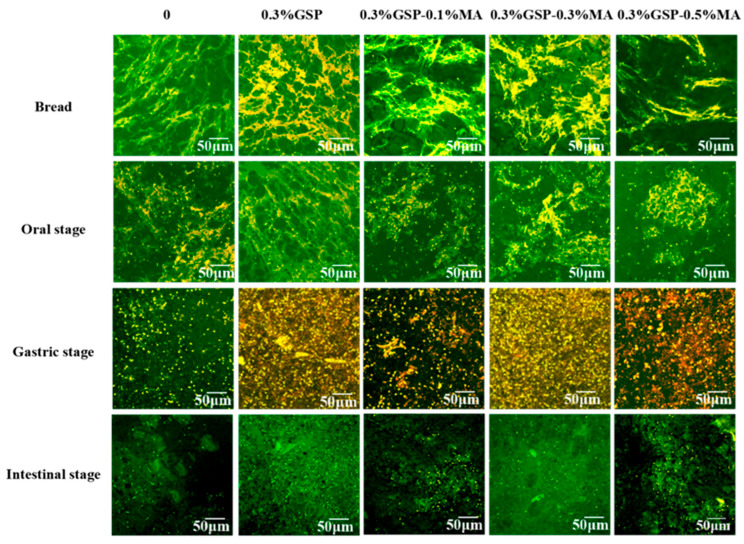
Monitoring the digestive process of bread prepared with varying ratios of GSP and MA through confocal laser scanning microscopy at the oral, gastric, and intestinal stages, with abbreviations referenced in Figure 1.

Confocal laser scanning microscopy (CLSM) images of bread at different digestion stages are documented in Figure 5. The series of images shows the microscopic structure of bread from the three stages of digestion, from the oral to the intestinal phases. The starch granules in all bread absorbed water and expanded significantly before digestion, forming swollen granular structures (Figure 5). The gluten structure of GSP-containing breads was dense, and this matrix persisted throughout the oral and gastric phases, protecting starch from an enzymatic attack through a physical barrier. In the presence of MA, the gluten structure was looser, and more starch particles were exposed at the increased 0.5% MA concentration. At the oral stage, the starch in the bread that was treated by digestive enzymes was disrupted and released to a low degree, and the protein structure loosened [40]. At the gastric stage, different degrees of gluten and starch decomposition were observed, and gluten was broken down into small fragments [41]. GSP enhanced the gluten strength and resulted in smaller gluten fragments, but numerous starch particles were still enclosed. In the small intestine stage, gluten protein was almost completely digested, and no significant difference was found among different samples. However, starch was digested to different degrees, consistent with the glucose release data after digestion. Breads with 0.3%GSP and 0.3%GSP–0.1%MA showed less starch digestion. This finding may be related to the fact that GSP and MA inhibited the activity of digestive enzymes. Likewise, the presence of GSP may interfere with the starch–gluten interaction and ultimately inhibit starch digestion [7].

### 3.6. Inhibitory Effects of Proanthocyanidins and MA on the Activities of α-Amylase and α-Glucosidase

The α-amylase inhibition rate increased notably in the presence of GSP and MA, reaching 57.7% at 0.3% GSP–0.5% MA, which was 10.3% higher than in the control group (Table 3). In contrast, α-glucosidase inhibition remained stable (approximately 75%), indicating that the inhibitor selectively targets α-amylase for starch hydrolysis [42].

**Figure 6:**
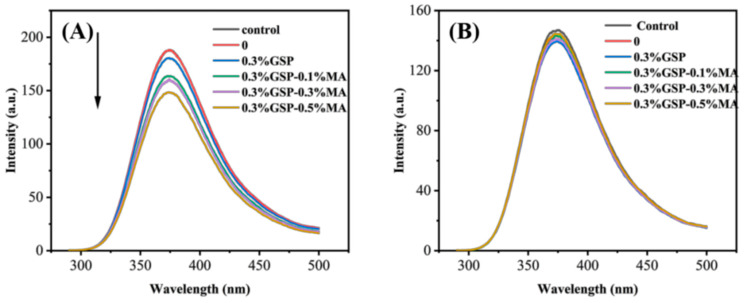
Fluorescence quenching of α-amylase (**A**) and α-glucosidase (**B**) in bread samples compounded with wheat starch, 0.3%GSP, 0.3% Gsp–0.1% MA, 0.3% GSP–0.3% MA, and 0.3% GSP–0.5% MA. Abbreviations as in Figure 1.

GSP and MA influenced the fluorescence intensities of α-amylase and α-glucosidase in the bread system (Figure 6). The fluorescence-quenching effects of GSP and MA on enzymes were compared by monitoring the maximum fluorescence intensity in the emission spectrum of tryptophan residue indole groups at 280 nm excitation. Including GSP resulted in a slight red shift from 373 nm to 374 nm. The fluorescence intensity also decreased gradually with the addition of GSP and MA. This finding indicates that the hydrogen bonding and hydrophobic interactions occurred between GSP and MA and tryptophan residues, thereby inducing conformational changes and reducing catalytic efficiency [43]. The maximum emission of α-glucosidase showed a slight blue shift, from 377 nm to 374 nm. However, fluorescence intensity did not change significantly, consistent with the results of the enzyme-activity inhibition rate. Thus, the effects observed of GSP and MA were most likely due to binding to α-amylase.

## 4. Conclusions

This study systematically investigates the effects of grape seed proanthocyanidins (GSP) and malic acid (MA) on the structural properties of wheat starch and the in vitro digestion behavior of bread. The combination of GSP and MA increased the molecular order of starch. WAXS data showed that the combination of GSP and MA effectively reduced the crystallinity of the starch matrix. Bread made with GSP and MA still had a large number of undigested starch granules after digestion, and it showed suppressed glucose release after digestion as compared to the control. Enzyme fluorescence spectral analysis showed that both GSP and MA had a notable fluorescence-quenching effect on α-amylase and that both inhibited the activity of α-amylase. However, no significant quenching was observed for α-glucosidase. These findings indicate that the synergistic action of GSP and MA modulates starch digestibility through combined structural and enzymatic mechanisms, offering a practical and scalable approach for the development of functional staple foods with reduced glycemic impact, thereby contributing to the growing demand for health-oriented innovations in the cereal-based food industry.

## Figures and Tables

**Table 1:** Short-range structural order of raw wheat starch granules as monitored by FTIR, 0.1%GSP, 0.3%GSP, 0.5%GSP, 0.3%GSP-0.1%MA, 0.3%GSP-0.3%MA, and 0.3%GSP-0.5%MA.

Sample	1047/1022 cm^−1^	1022/995 cm^−1^
Wheat starch	0.877	1.059
0.1%GSP wheat starch	0.878	1.060
0.3%GSP wheat starch	0.881	1.080
0.5%GSP wheat starch	0.877	1.070
0.3%GSP-0.1%MA wheat starch	0.881	1.096
0.3%GSP-0.3%MA wheat starch	0.886	1.070
0.3%GSP-0.5%MA wheat starch	0.884	1.078

**Table 2:** Full width at half-maxima (FWHM) for the 480 cm^−1^ Raman peak of raw wheat starch, indicating structural change in the starch in dependence on additives. Percentages of GSP and MA are indicated. Abbreviations as in Figure 1.

Wheat Starch	0	0.1%GSP	0.3%GSP	0.5%GSP	0.3%GSP-0.1%MA	0.3%GSP-0.3%MA	0.3%GSP-0.5%MA
FWMH at 480 cm^−1^	29.09	27.35	27.15	29.63	28.90	27.68	29.52

**Table 3:** Inhibitory effects on α-amylase and α-glucosidase activities of the addition of 0.3%GSP, 0.3%GSP-0.1%MA, 0.3%GSP-0.3%MA, and 0.3%GSP-0.5%MA in bread samples prepared with wheat starch. Different letters indicate significant difference at *p* = 0.05.

Sample	α-Amylase (%)	α-Glucosinase (%)
0	52.2 ± 0.8 ^d^	75.3 ± 0.3 ^a^
0.3%GSP	54.3 ± 0.8 ^b^	75.0 ± 0.4 ^a^
0.3%GSP-0.1%MA	52.8 ± 0.8 ^cd^	75.1 ± 0.1 ^a^
0.3%GSP-0.3%MA	54.2 ± 0.6 ^bc^	75.4 ± 0.2 ^a^
0.3%GSP-0.5%MA	57.7 ± 1.3 ^a^	75.1 ± 0.7 ^a^

## Data Availability

The original contributions presented in this study are included in the article. Further inquiries can be directed to the corresponding authors.
